# On predicting particle capture rates in aquatic ecosystems

**DOI:** 10.1371/journal.pone.0261400

**Published:** 2021-12-22

**Authors:** Alexis Espinosa-Gayosso, Marco Ghisalberti, Jeff Shimeta, Gregory N. Ivey

**Affiliations:** 1 School of Civil, Environmental and Mining Engineering, The University of Western Australia, Perth, WA, Australia; 2 Oceans Graduate School, The University of Western Australia, Perth, WA, Australia; 3 Centre for Environmental Sustainability and Remediation, School of Science, RMIT University, Bundoora, VIC, Australia; University of New South Wales, AUSTRALIA

## Abstract

Recent advances in understanding the capture of moving suspended particles in aquatic ecosystems have opened up new possibilities for predicting rates of suspension feeding, larval settlement, seagrass pollination and sediment removal. Drawing on results from both highly-resolved computational fluid dynamics (CFD) simulations and existing experimental data, we quantify the controlling influence of flow velocity, particle size and collector size on rates of contact between suspended particles and biological collectors over the parameter space characterising a diverse range of aquatic ecosystems. As distinct from assumptions in previous modeling studies, the functional relationships describing capture are highly variable. Contact rates can vary in opposing directions in response to changes in collector size, an organism’s size, the size of particles being intercepted (related to diet in the case of suspension feeders), and the flow strength. Contact rates shift from decreasing to increasing with collector diameter when particles become relatively large and there is vortex shedding in the collector wake. And in some ranges of the ecologically relevant parameter space, contact rates do not increase strongly with velocity or particle size. The understanding of these complex dependencies allows us to reformulate some hypotheses of selection pressure on the physiology and ecology of aquatic organisms. We discuss the benefits and limitations of CFD tools in predicting rates of particle capture in aquatic ecosystems. Finally, across the complete parameter space relevant to real aquatic ecosystems, all quantitative estimates of particle capture from our model are provided here.

## Introduction

**Fig 1:**
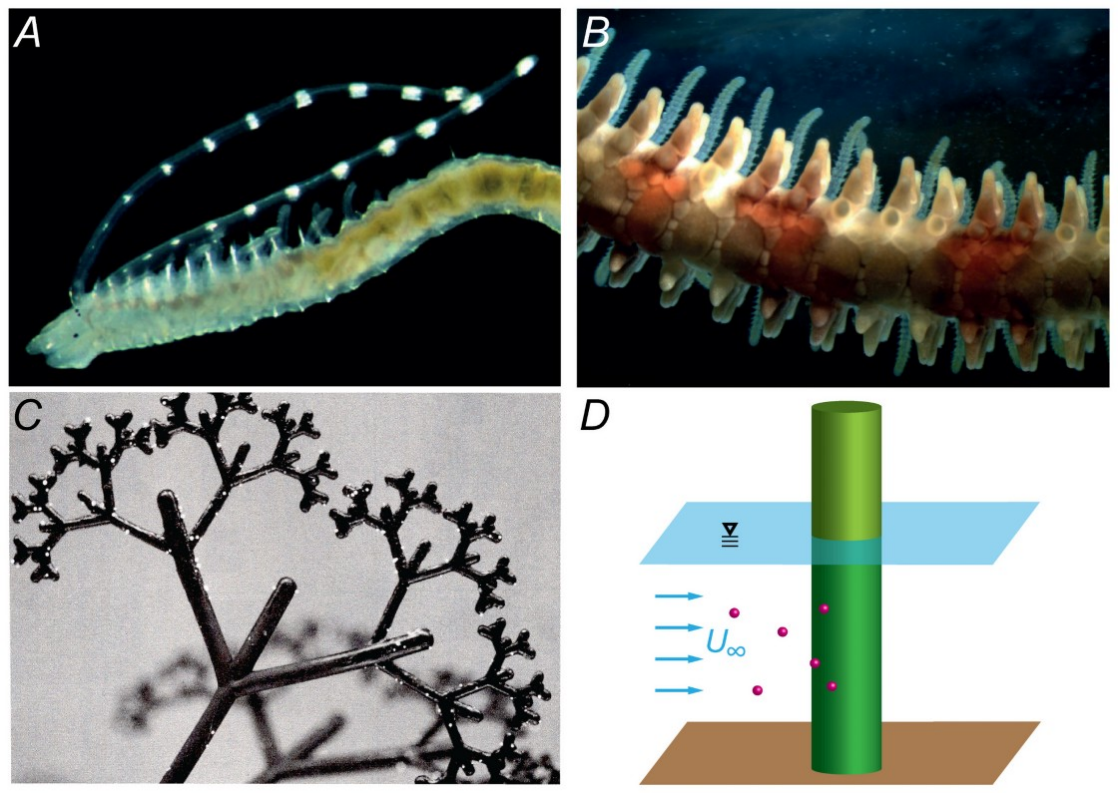
Examples of the particle collectors employed in laboratory and numerical experiments. (***A***) Pair of long palps (visible as tentacles with white pigment spots) of a spionid polychaete (e.g. [7, 17], photograph by L. Harris). (***B***) Multiple tube feet in the arm of a brittle star (e.g. [5], photograph by J. Sones). (***C***) A branched model collector emulating filamentous red algae [13]. (***D***) A rigid cylinder employed as an archetypal collector structure (e.g. [14]).

The capture of suspended particles by aquatic ‘collectors’ is a critically-important process governing the health, productivity and propagation of some of the most productive and biodiverse ecosystems on the planet [1]. Key processes that rely on the capture of particles in suspension include: the feeding on seston by corals and other suspension feeders [2–7] (Fig 1*A* and 1*B*), the uptake of microplastic particles [8, 9], the pollination of seagrasses [10, 11], the settlement of larvae on solid substrates such as filamentous algae or kelp [12, 13] (Fig 1*C*), and sediment removal by aquatic vegetation [14–16]. Despite its significance, particle capture in aquatic ecosystems remains poorly characterized, due largely to the dynamic complexity of flow-particle-collector interactions.

Recent advances in modeling and observation for certain types of particle collectors have yielded insights into their interactions with flow fields that influence particle capture. For example, three-dimensional modeling and experimental flow visualization around pulsating sea jellies have explained behavioural influences on vortex dynamics and prey clearance [18, 19]. The kinematics of a novel means of particle concentration, referred to as ricochet separation, has been described in manta rays [20]. The effects of collector motion, such as moving feeding appendages or vegetation under wave oscillation, on particle capture have been modeled and measured [21–23]. Particle motility, such as that of zooplankton, has also been shown to allow the evasion of capture, reducing particle capture rates [24]. Sediment trapping by canopies of aquatic vegetation has been modeled in terms of shoot geometry and density, flow fields, and their interactions with particles, and these effects measured in flume experiments [15, 16]. While such studies address some of the real-world complexities of ecosystem particle capture, widely applicable modelling that accurately predicts the crucial step of physical contact between particles and collectors for a broad range of organisms and ecosystems remains elusive.

The first step in particle capture is contact between particle and collector, and the particle must be subsequently retained by the collector for capture to occur. This retention is often made possible by adhesive layers on the particle or collector surfaces generated by, for example, mucus [6] or the periphyton layer of aquatic vegetation [25]. Retention may also be engendered by electrostatic forces [6] or with the aid of additional structures such as nematocysts [7]. It is acknowledged that retention is a necessary aspect of particle capture, varying widely with the biological, chemical and physical characteristics of particle and collector. This study, though, focuses on quantitative description of the initial (and essential) contact process to provide a first-order indication of how ecosystem particle capture rates will vary with key system characteristics.

**Fig 2:**
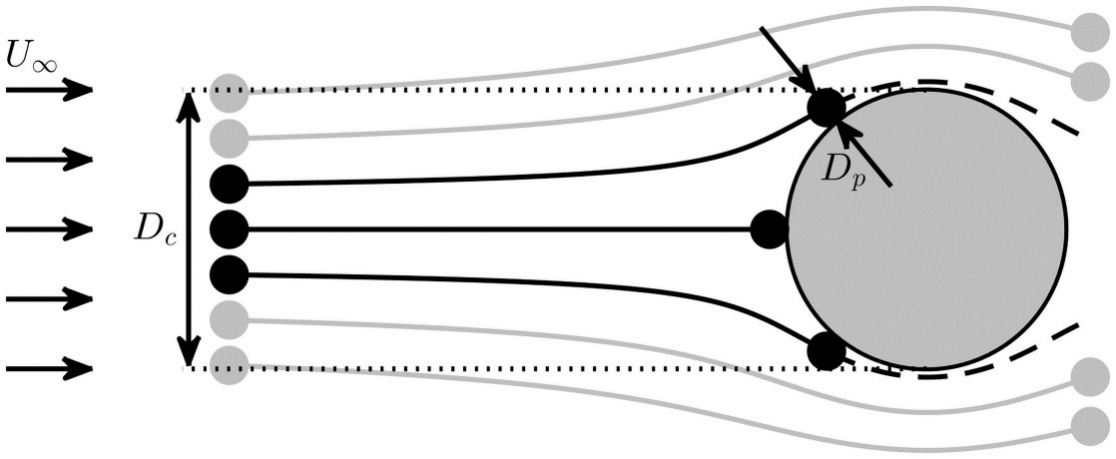
Plan view of particle capture through direct interception by a collector with diameter *D*_*c*_ in an upstream flow velocity *U*_∞_. Contact occurs when particle centers come within one particle radius of the collector surface (black particles). The contact efficiency of the collector (*η*) is the fraction of approaching particles that make contact (Eq 2); ‘approaching’ particles are those that flow through an upstream window of width *D*_*c*_ and height *h*_*c*_ (into the page).

There are three fundamental mechanisms of particle contact with vertical collectors [26]: (i) direct interception, where particle centers follow fluid pathlines and contact occurs because of the finite particle size (Fig 2); (ii) inertial impaction, where particle inertia causes a deviation of particle trajectories from fluid pathlines and creates contact with the collector; and (iii) diffusional deposition, where particle-collector contact is driven by random particle motions (such as Brownian motion). Capture due to diffusional deposition is typically negligible compared with direct interception, only becoming important for particles with diameters O(μm) or less [27]. Inertial impaction tends to be significant only for large particles that are much denser than water (for example, suspended sediment particles with a size exceeding roughly 5% of the collector diameter, [28]). Direct interception is thus typically the dominant mechanism of contact between particles and collectors in aquatic systems [3, 6, 14, 28–30], and is the focus of this study.

This paper provides a quantitative understanding of the particle capture process in real aquatic ecosystems by accounting for its complex variation with the ambient flow velocity, the size of the particles being captured, and the size of the collectors. The subsequent handling of particles in contact with collectors and the structural resistance of the collector can also be important. These factors collectively determine the optimal combination to maximise capture, an optimum that may have led to the evolution of the size of collecting structures in aquatic ecosystems.

### Modelling framework

For the problem to be tractable, many simplifications are typically employed in experimental (e.g [13, 14]), analytical (e.g [26]) and numerical (e.g [28, 31, 32]) models of particle capture. Models typically idealize collectors as cylinders (Fig 1*D*), while suspended particles are idealized as spheres (Fig 2). In truth, real biological collectors are undoubtedly more structurally complex than the canonical cylinder form examined here. However, the philosophy of the approach adopted here is to focus on development of a generalisable model to both (a) confirm the role of computational fluid dynamics in generating quantitative predictions of ecosystem particle capture and (b) greatly improve our understanding of the sensitivity of that process to ecosystem characteristics. We demonstrate through comparison of results from this generalisable model with experimental observations of real, complex geometries (as shown in Fig 1*A*–*1C*) that this approach indeed yields a robust understanding of ecosystem particle capture and the drivers of changes to the rates of this process.

**Table 1:** List of symbols.

Symbols	Description
*C*_*p*_	ambient particle concentration
*CR*	contact rate
*D*_*c*_	collector diameter
*D*_*p*_	particle diameter
*F*_*p*_	particle flux approaching the cylindrical collector (= *C*_*p*_ *U*_∞_ *h*_*c*_ *D*_*c*_)
*h*_*c*_	collector height
*k*_1_, *k*_2_	constants of proportionality
*r*_*p*_	particle size ratio (= *D*_*p*_/*D*_*c*_)
Re	Reynolds number (= *U*_∞_ *D*_*c*_/*ν*)
*U*_∞_	uniform upstream flow velocity
*α*	exponent ruling variation of *η* with Re
*β*	exponent ruling variation of *η* with Re and of *C η R* with *D*_*p*_
*γ*	exponent ruling variation of *C* η *R* with *U*_∞_ (≡ 1 + *α*)
*η*	contact efficiency (= *C* η *R*/*F*_*p*_)
*ν*	kinematic fluid viscosity
*δ*	exponent ruling variation of *C* η *R* with *D*_*c*_ (≡ 1 + *α* − *β*)
O	symbol indicating order of magnitude
≡	is identical to
≈	is approximately equal to
∝	is proportional to
∼	is of order

Three quantities are needed to describe the extent of particle contact with a collector: (i) the flux of particles approaching the collector, (ii) the rate at which particles contact the collector, and (iii) the fraction of approaching particles that contact the collector, termed the contact efficiency. ‘Approaching’ particles are defined as those that pass through the upstream projected area of the collector which, for a cylindrical collector, is equal to the product of its diameter *D*_*c*_ and height *h*_*c*_ (Fig 2). (A list of all symbols is provided in Table 1). The particle flux approaching a cylindrical collector (*F*_*p*_) is equal to the product of the ambient particle concentration (*C*_*p*_) and the volumetric fluid flux through this projected area; i.e.

$$\begin{array}{c} F_{p}=C_{p}U_{\infty }h_{c}D_{c}, \end{array}$$ (1)

where *U*_∞_ is the upstream flow velocity (Fig 2). The contact rate (*CR*) represents the number of particles that contact the collector surface per unit time. The contact efficiency (*η*) represents the fraction of approaching particles that contact the collector:

$$\begin{array}{c} \eta =\frac{C\;R}{F_{p}}. \end{array}$$ (2)

Much of the research in this area has focused on developing tools for estimating *η* (e.g. [3, 14, 26, 28, 31, 32]). In many aquatic applications, the contact rate is the main quantity of interest [6] and is typically evaluated from Eq (2) after both *η* and *F*_*p*_ have been determined. In the case of direct interception dominance, the contact efficiency of a rigid cylindrical collector depends on two dimensionless parameters:

$$\begin{array}{c} \eta =\eta (\text{Re},r_{p}), \end{array}$$ (3)

[31] where *r*_*p*_ = *D*_*p*_/*D*_*c*_ is the particle size ratio (with *D*_*p*_ the particle diameter), and Re = *U*_∞_ *D*_*c*_/*ν* is the collector Reynolds number (with *ν* the kinematic fluid viscosity). These dimensionless parameters span orders of magnitude in value across the range of collectors in aquatic ecosystems (Table 2).

**Table 2:** Typical values of dimensionless parameters Re and *r*_*p*_ in aquatic ecosystems where particle capture is a fundamentally-important process.

Collector	Particle	*D*_*c*_ [*μ*m]	*D*_*p*_ [*μ*m]	Re^†^	*r*_*p*_	Reference
1. Cnidarian tentacle	Plankton	40–2000	10–100	0.04–400	0.005–2.5	[3, 6]
2. Echinoderm tube foot	Plankton	30–300	10–100	0.03–60	0.03–3	[3, 6]
3. Polychaete palp	Plankton	50–100	10–100	0.05–20	0.1–2	[3, 6]
4. Red algae branch	Invertebrate larvae	600–1700	125–300	0.6–340	0.07–0.5	[13]
5. Seagrass stigma	Pollen	100	100	0.1–20	up to O(10)	[10]
6. Wetland vegetation	Sediment	2500–8000	100–300	2.5–1600	0.01–0.15	[14]

^†^ Values of Re were estimated assuming upstream velocities (*U*_∞_) between 0.1 and 20 cm s^−1^ [3, 6, 31]

### Existing predictive tools

Until recently, the only tools available for predicting contact efficiency in aquatic ecosystems were based on analytical formulations, each developed for limiting values of the governing parameters. Such formulations are restricted to very small particle sizes (i.e. *r*_*p*_ ≪ 1) at either very low Reynolds number (the creeping flow limit, Re ≪ 1) [33] or very large Reynolds number (Re ≫ 1). For example, in the case of creeping flow (low Reynolds number) [33] found that

$$\begin{array}{c} \eta =(\frac{1}{2.0-\text{Re}}){r_{p}}^{2}\;\text{for}\;\text{Re}\ll 1\;\left(\text{and}\;r_{p}\ll 1\right). \end{array}$$ (4)

In contrast, boundary layer theory (large Reynolds number) predicts that

$$\begin{array}{c} \eta =k_{1}{\text{Re}}^{0.5}{r_{p}}^{2}\;\text{for}\;\text{Re}\gg 1\;\left(\text{and}\;r_{p}\ll 1\right), \end{array}$$ (5)

[26, 34], with *k*_1_ a constant of proportionality, a relationship confirmed by [35]. Not only are Eqs (4) and (5) very different, the Reynolds numbers and particle size ratios encountered in aquatic ecosystems rarely conform to such limiting behaviours. They fall within ranges spanning orders of magnitude (i.e. $O(10^{-1})<\text{Re}<O(10^{3})$ and $O(10^{-1})<r_{p}<O(1-10)$, Table 2), ranges over which these analytical formulations are of limited utility [3, 6, 31].

Formulations for estimating *η* in the parameter space relevant to aquatic ecosystems have only recently been developed [3, 14, 31, 35]. For example, [14] followed an engineering approach in fitting experimental measurements of capture by a cylindrical collector (as in Fig 1*D*) to a formulation with the same dependencies as Eq (5) but with the exponents left as curve-fitting parameters. This resulted in the empirical expression

$$\begin{array}{c} \eta =0.224{\text{Re}}^{0.718}{r_{p}}^{2.08}, \end{array}$$ (6)

an expression valid over the relevant, but not comprehensive, ranges of 38 < Re < 486 and *r*_*p*_ < 0.03. Similarly empirical expressions for capture efficiency were provided by [3], albeit limited to low Reynolds number (Re < 10).

This research team has previously developed a state-of-the-art computational fluid dynamics (CFD) model of the flow-particle-collector interaction to determine particle contact over the *entire* parameter space relevant to aquatic ecosystems (namely, 0 < Re ≤ 1000 and 0 < *r*_*p*_ ≤ 1.5) [28, 31, 32]. There is excellent agreement of model results with analytical predictions at low Re, and with experiment at higher Re. As the dependence of contact efficiency on the dimensionless parameters varies significantly over the parameter space, no one expression was able to accurately and fully describe the contact efficiency in aquatic systems. Consequently, results were reported in a series of diagrams, such as Fig A in S1 Dataset. Here, we re-formulate the results from this CFD modelling approach to:

1. Generate a comprehensive dataset (provided in S1 Dataset) of particle capture efficiency in order to provide a digital tool to enable particle capture prediction. Rather than just simplified behavior in the limits of vanishing particle size and very small/very large Reynolds number, we focus on particle capture across the full parameter space of aquatic ecosystems.
2. Demonstrate the extensive variation of particle capture rates experienced by real biological collectors. We highlight the limitations of relationships that present a fixed dependence of capture on flow velocity, particle size and collector size in order to provide the most comprehensive picture of particle capture variation.
3. Reconcile the full range of disparate experimental observations of ecosystem particle capture. Across a wide and relevant range of system variables, the model is shown to accurately predict the variation of particle capture rates observed in experiments involving real and model collectors, allowing for the first time quantitative prediction of particle capture in *real* ecosystems.

## Materials and methods

Analysis of the effects of system variables on particle capture was undertaken with the aid of output from the validated numerical model developed by Espinosa-Gayosso *et al.* [28, 31, 32] and existing experimental data of particle capture by real suspension feeders [5, 7, 17] and synthetic laboratory structures [13, 14]. Numerical model predictions were compared directly to these experimental results to demonstrate the accuracy of the numerical approach and its applicability to complex aquatic ecosystems.

### Isolating the impact of system variables on particle contact rate

The impact that dimensional variables such as flow velocity, particle size and collector size have on contact rate is embedded within the influence of the dimensionless parameters Re and *r*_*p*_ in Eq (3). With the complex forms that Eq (3) can take, it is not always intuitive to understand the impact of each of these system variables. For example, the collector diameter *D*_*c*_ influences both the particle size ratio and collector Reynolds number, providing complex control on the overall contact rate. While no single formulation describes contact due to direct interception over the entire parameter space of interest, it is nevertheless useful to consider a generalized form of the theoretical expression for contact efficiency through direct interception at high Reynolds number (Eq (5)), namely:

$$\begin{array}{c} \eta =k_{2}{\text{Re}}^{\alpha }{r_{p}}^{\beta }\;. \end{array}$$ (7)

Eq (7) can be rewritten as

$$\begin{array}{c} \eta =k_{2}{\left(\frac{U_{\infty }D_{c}}{\nu }\right)}^{\alpha }{\left(\frac{D_{p}}{D_{c}}\right)}^{\beta }=k_{2}\nu^{-\alpha }{U_{\infty }}^{\alpha }{D_{p}}^{\beta }{D_{c}}^{\alpha -\beta }\;. \end{array}$$ (8)

Hence, from Eqs (1) and (2), the contact rate can be expressed as:

$$\begin{array}{cc} C\;R & =\eta F_{p}\\ & =k_{2}C_{p}h_{c}\nu^{-\alpha }{U_{\infty }}^{1+\alpha }{D_{p}}^{\beta }{D_{c}}^{1+\alpha -\beta }\\ & \equiv k_{2}C_{p}h_{c}\nu^{-\alpha }{U_{\infty }}^{\gamma }{D_{p}}^{\beta }{D_{c}}^{\delta }\;. \end{array}$$ (9)

The exponents *α*, *γ* ≡ 1 + *α*, *β* and *δ* ≡ 1 + *α* − *β* on the right-hand side of Eq (9) are considered variable over the parameter space. However, ‘local’ values of these exponents can be evaluated to explain the effects of key variables on particle capture. It will be seen that local values of the exponents vary widely (and even change sign) as system conditions change.

### Numerical model output

The model output used to analyze the effects of key variables on contact rate comes from a series of Direct Numerical Simulations (DNS) of flow around a rigid cylindrical object in the ranges 0.01 ≤ Re ≤ 1000 and 0 < *r*_*p*_ ≤ 1.5. These ranges are of the most relevance to aquatic ecosystems (Table 2). This Re range covers several regimes of flow around a cylindrical collector: (i) a steady two-dimensional flow regime (Re ≤ 47); (ii) an unsteady two-dimensional vortex-shedding regime (47 < Re ≤ 180); (iii) a first form of unsteady three-dimensional vortex-shedding regime (180 < Re ≤ 260); and (iv) a second form of three-dimensional vortex-shedding regime (260 < Re ≤ 1000) [32, 36]. At least 10 simulations per logarithmic decade of Re were performed here.

By solving the governing (Navier-Stokes) equations with mesh refinement, the numerical simulations fully resolved the typically time-varying flow observed around the collector down to the smallest physical and particle path scale around the collector [31, 32]. The velocity boundary conditions applied to the edges of the numerical domain were: (i) a steady uniform free-stream at the upstream boundary, (ii) no-flux, free-slip conditions at the lateral boundaries, and (iii) a zero-gradient condition perpendicular to the downstream boundary. A no-slip, no-flux boundary condition was applied to the cylindrical collector surface. For three-dimensional simulations (Re ≥ 180), cyclic conditions were applied at the top and bottom (axial) boundaries [31, 32]. For the pressure field, a zero-gradient condition was applied at all boundaries except the outlet, where a fixed value of pressure was set, and at the cyclic axial boundaries [31, 32]. Domain edges were chosen to be many cylinder scales from the collector in order to avoid numerical blockage effects [31, 37]. The length of the domain in the axial direction was large enough to allow the most unstable wavelengths in the three-dimensional vortex shedding regime to develop properly [32, 38].

As direct interception is the mechanism of contact considered here, particles are considered to behave as perfect tracers, meaning that the centers of neutrally-buoyant particles follow fluid pathlines exactly. Using the same approach as other successful numerical and theoretical studies [3, 26, 39], we also assume that there is no influence of the particles on the flow and there is negligible particle-particle interaction (thereby assuming low particle concentrations). These assumptions imply that the influences of several other forces on the particles, such as lift induced by shear, ‘short range’ (such as Van der Waals or electrical double-layer) forces and hydrodynamic repulsion to contact, are considered of lower order. Finally, the finite-size spherical particles are considered captured upon contact with the collector (Fig 2).

It is very important to note that the particle dynamics considered here, together with the numerical settings for the DNS, have previously been carefully validated against experimental data in the ranges of interest in three previous publications. In particular, the difference between observation and model in mean contact efficiency was less than 3%, with numerical predictions always within the ranges of experimental error; in [31] the validation was against low Re, while in [32] the validation was for higher Re. Furthermore, in [28] it was confirmed theoretically and numerically that for the Re range presented here, neutrally buoyant particles indeed behave as perfect tracers and the effects of added mass and velocity gradient effects can be neglected. The reader should refer to these prior publications for full details of the numerical methodology and a description of the model validation.

The full set of output generated by the validated predictive model is provided, for the first time, in the digital tool in S1 Dataset. This data set permits accurate quantitative prediction of contact rate (*CR*) and contact efficiency (*η*) of neutrally-buoyant suspended particles with a single biological collector. The input required to obtain contact rate and efficiency estimates consists of: the flow velocity (*U*_∞_), particle and collector diameters (*D*_*p*_ and *D*_*c*_, respectively), collector height (*h*_*c*_), the concentration of particles in suspension (*C*_*p*_) and fluid kinematic viscosity (*ν*). This repository is far more comprehensive than simply the numerical results presented here, fully spanning the ranges 0 ≤ Re ≤ 1000 and 0 ≤ *r*_*p*_ ≤ 1.5 and relevant to the full range of biological collectors in aquatic ecosystems (e.g. Table 1). Analytical results from creeping flow theory were utilised to complete the dynamical desciptions for very low Re. In previous work, the contact efficiency (*η*) was typically provided in graphical form, as shown in Fig A in S1 Dataset.

Values of *α* and *β* in Eq (7) were then determined by evaluating changes in contact efficiency between neighboring points (‘1’ and ‘2’) in the (log-log) Re-*r*_*p*_ space of the model output, i.e.:

$$\begin{array}{c} \alpha =\frac{\text{ln}\frac{\eta_{1}}{\eta_{2}}}{\text{ln}\frac{{\text{Re}}_{1}}{{\text{Re}}_{2}}}\;\;\left(r_{p1}=r_{p2}\right)\; \end{array}$$ (10)

$$\begin{array}{c} \beta =\frac{\text{ln}\frac{\eta_{1}}{\eta_{2}}}{\text{ln}\frac{r_{p1}}{r_{p2}}}\;\;\left({\text{Re}}_{1}={\text{Re}}_{2}\right). \end{array}$$ (11)

The definitions *γ* ≡ 1 + *α* and *δ* ≡ 1 + *α* − *β* were then used to quantify all exponents in Eq (9), and thus characterise the influence of all system variables on particle capture rate.

### Analysis of experimental data

As shown in Table 3, the experimental studies chosen for the analysis of particle contact rates in aquatic ecosystems cover a range of different collectors and configurations (including all those shown in Fig 1). These include data of particle capture by suspension feeders, such as brittle stars [5] and species of polychaetes [7, 17], larval capture and settlement on branched red-algae-type structures [13], and systematic laboratory studies of particle capture on rigid cylindrical collectors [14]. With the exception of the polychaete studies, the rate of particle capture (rather than contact) was measured. To allow comparison between model predictions and experimental data, perfect particle retention (i.e. equal rates of contact and capture) was assumed.

**Table 3:** Summary information of the experimental studies analyzed here.

Particle collector	*U*_∞_ [cms^−1^]	*D*_*c*_ [*μ*m]	*D*_*p*_ [*μ*m]	Re	*r*_*p*_	Reference	Plot marker
1. Spionid polychaetes	3–12	70, 120^†^	32	2.1–14	0.26–0.5	[17]	△
2. Brittle star	4	217	40–320	8.7	0.18–1.5	[5]	☆
3. Spionid polychaetes	1.3–9.1	60–200	32, 82	0.7–18	0.15–1.4	[7]	∇
4. Red-algae-type structure	5^†^	500–1700	200^†^	25–70	0.12–0.4	[13]	*
5. Single rigid cylinder	0.6–1.8	6350–25400	194	38–460	0.008–0.03	[14]	▫ (*D*_*c*_ = 6 mm)
							◊ (12 mm)
							○ (25 mm)

^†^These values were not reported in the original paper and needed to be estimated from other literature.

#### Suspension feeders: Spionid polychaetes and brittle star

The *effect of flow velocity* on the rate of particle contact with palps of the spionid polychaete *Polydora cornuta* was investigated in the laboratory experiment of [17] (Fig 1*A* & Row 1, Table 3). Juvenile and adult worms with different (but unreported) palp diameters were used; here, we assume palp diameters of 70 *μ*m for juveniles and 120 *μ*m for adults, the mean diameters reported for polychaetes in [7]. The exposed collector length (*h*_*c*_) was also not reported; in establishing the prediction of the influence of flow velocity on contact rate, the exposed length was taken to be that which gave perfect model-experiment agreement for the experiment with the lowest flow velocity.

The *effect of particle size* was determined through an investigation of particle capture by the brittle star *Ophiopholis aculeata* [5] (Fig 1*B* & Row 2, Table 3), where the tube feet of the brittle star were identified as the main collecting structures. The water in the experimental flume was maintained at constant velocity and seeded with particles with a range of sizes (Table 3). Due to settling, the average size of particles in suspension decreased over the approximately 3 minutes of the experimental runs. This decrease was incorporated into model predictions by estimating the time-varying size distribution of particles in suspension, predicting particle capture counts within each bin of the size distribution during each time step, and summing the capture count within each bin over the duration of the experiment. The effect of particle size was also investigated in the flume experiment of [7] (Fig 1*A* & Row 3, Table 3), with palps of two species of spionid polychaetes (*Pseudopolydora paucibranchiata* and *Polydora kempi japonica*) as collectors. Suspended plastic beads of two diameters were used in varying ambient concentrations, with the ratio of the contact rates of the larger and smaller particles (*CR*_large_/*CR*_small_) reported.

#### Red-algae-type structures

The *effect of collector diameter* on contact rate was investigated in field measurements of the capture of bivalve spat by algae-type structures [13] (Fig 1*C* & Row 4, Table 3). The collectors had a branched structure (with branches having different diameters), mimicking filamentous red algae. Although the spat diameter and fluid velocity were not reported, here we use 212 *μ*m for the mean spat diameter and 5 cm s^−1^ for the mean velocity, values reported for a flume experiment in the same study which strove to emulate the field conditions. As the ambient concentration of bivalve spat was not reported, it was not possible to obtain model predictions of contact rate. Instead, experimental data are re-expressed as a ‘normalized’ contact rate; that is, the rate of contact with a collector of given diameter relative to that with the smallest collector employed. This allows direct comparison between experimental and numerical estimates of relative rates of contact.

#### Rigid cylinders

The *effects of flow velocity* and *collector diameter* on particle contact with rigid cylindrical collectors were investigated in the flume experiments of [14] (Fig 1*D* & Row 5, Table 3). The capture of plastic beads by cylindrical collectors, coated with a layer of adhesive grease, was quantified across a range of collector diameters and flow velocities.

## Results and discussion

### The variation of contact rate with flow velocity

**Fig 3:**
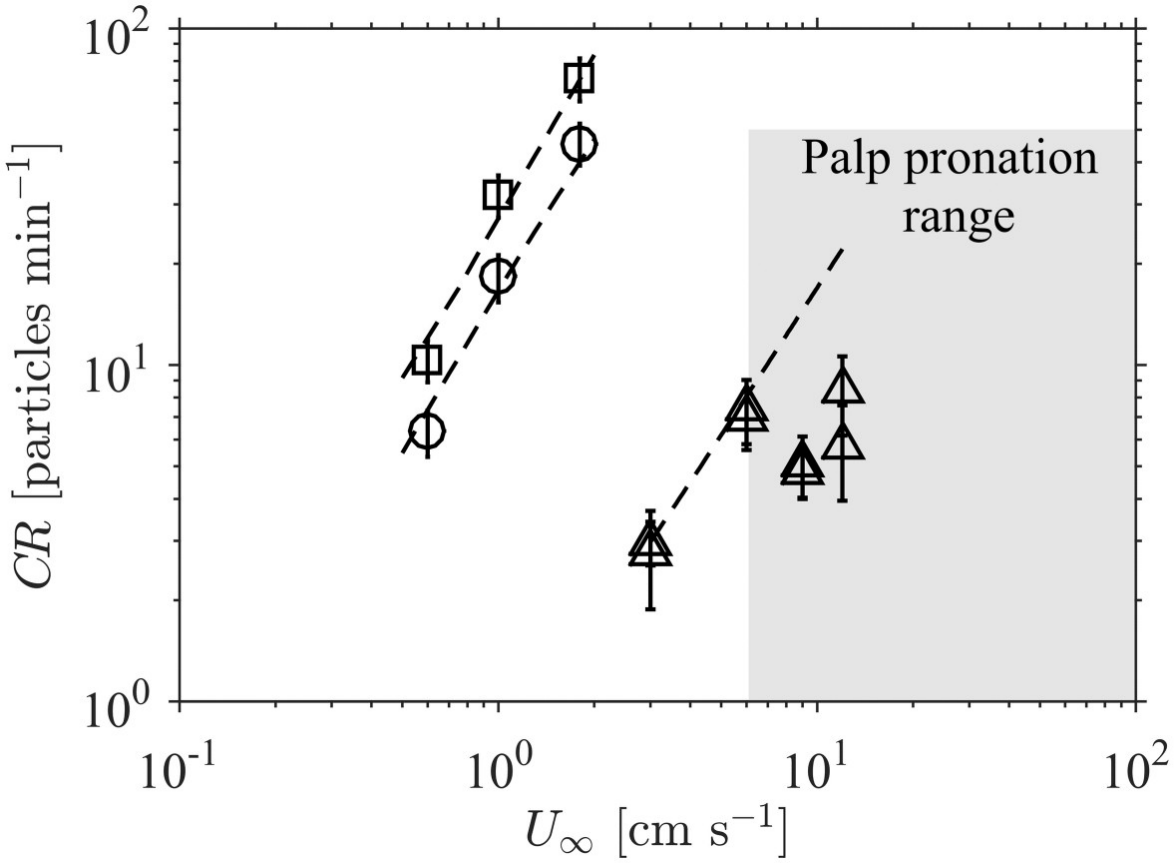
The impact of flow velocity on contact rate for both rigid and flexible collectors. Markers represent experimental data as per Table 2: single rigid cylinder with diameters of 6 mm (□) and 25 mm (○) [14] and flexible polychaetes (△) [17], with uncertainty as reported. Broken lines represent numerical model predictions of the contact rate variation. When the collectors do not experience flow-induced pronation (which, for the experimental polychaetes palps, occurred at velocities above 6 cm s^-1^, [17]), the predictive model performs excellently.

The effect of flow velocity (*U*_∞_) on contact rate (Fig 3) was determined with the aid of experiments involving a single rigid cylindrical collector [14] and various species of living polychaetes with flexible palps [17] (as in Fig 1*D* and 1*A*, respectively). The particle contact rate (*CR*) with rigid collectors (□ and ○ in Fig 3) increases strongly with the flow velocity. Importantly, the numerical model predicts this variation of *CR* excellently (i.e. within experimental uncertainty).

**Fig 4:**
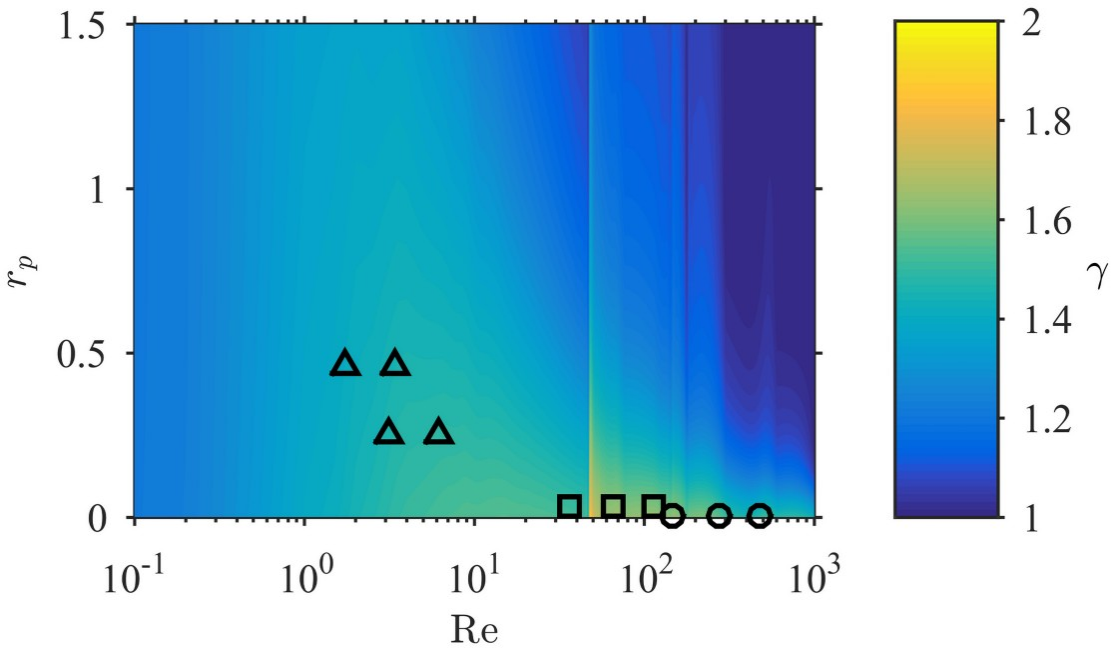
Model estimates of *γ*, which governs the dependence of contact rate on velocity ($C\;R\propto {U_{\infty }}^{\gamma }$), over the entire Re-*r*_*p*_ parameter space. The variability (1 ≤ *γ* ≤ 2) contrasts with the constant value of *γ* = 1.718 employed in Eq (6), highlighting the limitations of formulations with fixed exponents. The locations in the parameter space of experimental data from Fig 3 are shown; in the case of the flexible polychaetes (△), only data from the erect palps are shown here.

The increase of contact rate with flow velocity for rigid collectors is dictated by the local value of the exponent *γ* in Eq (9) (i.e. $C\;R\propto {U_{\infty }}^{\gamma }$) and its value is highly variable over the parameter space relevant to aquatic systems (Fig 4). The model shows that the exponent is consistently in the range 1 ≤ *γ* ≤ 2. Discontinuities appear because of fundamental changes in the nature of the flow; for example, at Re = 47, flow around a cylinder transitions from steady flow to unsteady vortex shedding [32]. At large Reynolds number (Re ≳ 500) and small particle sizes (*r_p_* ≲ 0.05), the numerical estimates are in agreement with boundary layer theory, which predicts *γ* = 1.5 (from *α* = 0.5 in Eq (5)). Over the parameter range of the experiments (markers in Fig 4), *γ* was within the range 1.4 − 2.0. These values explain the substantial increase of *CR* with *U*_∞_ in Fig 3. The constant value of *γ* = 1.718 in Eq (6), obtained from the experiments of [14], is an overestimate for most of the parameter space; this is particularly true for suspension feeders that commonly capture large particles ($r_{p}∼O\left(1\right)$), for which *γ* is much closer to 1. This serves to highlight the limitations of fixed-dependence (i.e. fixed exponent) formulations obtained over limited portions of the parameter space.

#### Impact of collector pronation

The effect of velocity on the rate of contact with *flexible* polychaetes is more complex. In low flow conditions (*U*_∞_ < 6 cm s^-1^), when the palps were erect, the contact rate increased with velocity at exactly the rate predicted by the numerical model that assumes collector rigidity (Fig 3). However, when the palps exhibited flow-induced pronation at velocities in excess of 6 cm s^-1^ ([17], the shaded region in Fig 3), contact rates fell significantly below rigid collector estimates. Many aquatic collectors, such as vegetation [21] or suspension feeders’ palps and cirri [4, 5, 7], have significant flexibility such that their geometry is modified by the flow. This deformation can be passive, such as bending due to hydraulic forces, or biologically active, such as polychaetes coiling their palps [17]. The deformation reduces the collector area projected into the flow, thereby creating a decrease in *CR* relative to that of the corresponding rigid collector. Thus, although the streamline compression that occurs in the neighborhood of the collector at higher velocities [3, 6, 31, 32] can have the positive effect of enhancing contact rates, Fig 3 demonstrates that collector deformation can constrain the benefits of strong flows for rates of suspension feeding, larval contact with substrata, and sediment trapping.

### The variation of contact rate with particle size

**Fig 5:**
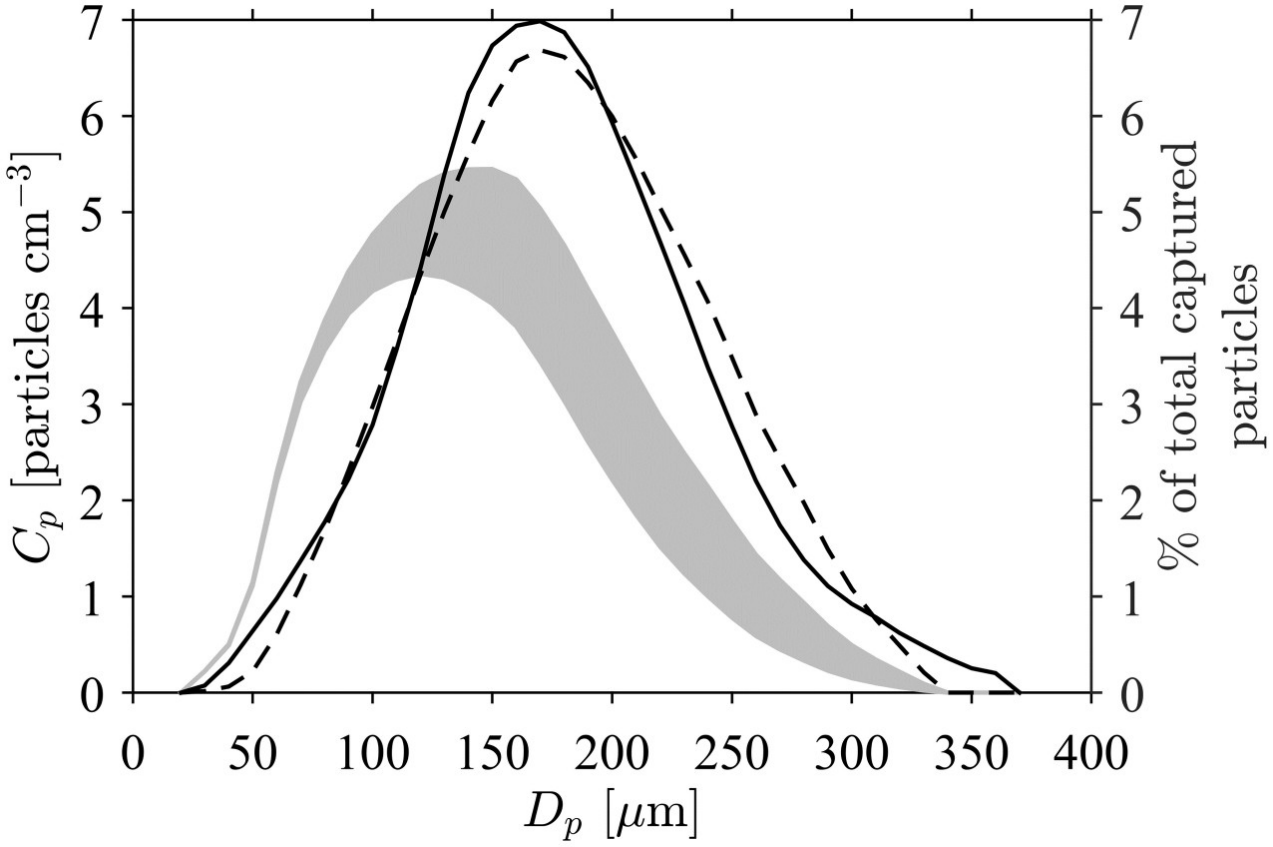
The discrepancy between size distributions of particles in suspension (gray band) and those captured by the tube feet of a brittle star (—) [5]. Settling during the experiment reduced both the concentration and average size of particles in suspension; the upper edge of the gray band represents the initial particle concentration distribution (on the left-hand axis) within 10 *μ*m bins, the lower edge the final distribution. Lines indicate (on the right-hand axis) the size distributions of *captured* particles: (i) observed experimentally in the bolus of the brittle star (—) and (ii) predicted by the numerical model (- - -). The greater peak particle diameter in the distribution of captured particles indicates an increasing contact rate with particle size.

The effect of particle size (*D*_*p*_) on contact rate (Fig 5) was analysed with the aid of the experimental measurements of particle capture by the tube feet of a brittle star [5]. Particles in suspension had a wide size distribution; the initial distribution had a broad peak around *D*_*p*_ = 150 *μ*m but, due to settling, this peak value decreased to approximately 110 *μ*m by the end of the experiment. The size distribution of particles captured by the tube feet of the brittle star is clearly distinct from that in suspension, showing a clear bias towards larger particles and a peak at *D*_*p*_ ≈ 170 *μ*m. This is a clear indication that larger particles experience higher rates of contact. The numerical model predicts the size distribution of captured particles, and its distinction from that of particles in suspension, very closely (Fig 5).

**Fig 6:**
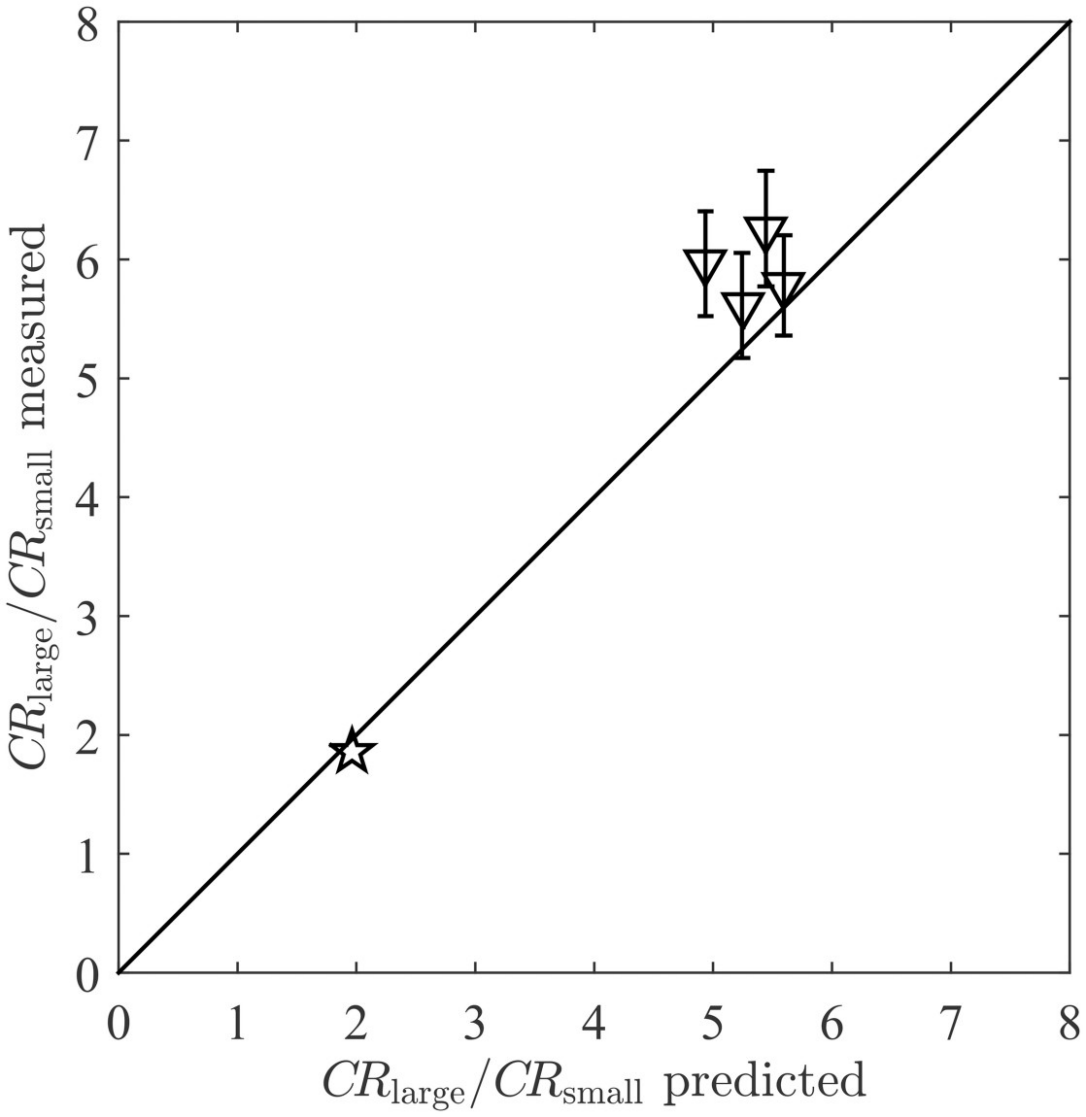
Comparison of model predictions with experimental measurements of the ratio of contact rates of larger and smaller particles in suspension (*CR*_large_/*CR*_small_). Markers represent the average of the four polychaetes experiments of [7] (∇) with *D*_*p*,large_ ≈ 82*μ*m and *D*_*p*,small_ ≈ 32*μ*m (error as reported), and the brittle star experiment of [5] (☆) with *D*_*p*,large_ ≈ 170*μ*m and *D*_*p*,small_ ≈ 110*μ*m. The solid line represents perfect agreement.

The increase in contact rate with particle size is also demonstrated by the experiments of [7]. In that study, contact rates of particles of two different sizes (*D*_*p*,large_ = 82*μ*m, *D*_*p*,small_ = 32*μ*m) with the palps of two types of spionid polychaetes were measured. The contact rates of the larger (*CR*_large_) and smaller particles (*CR*_small_) were not reported individually, but the ratio of the two (*CR*_large_/*CR*_small_) was reported across a range of velocities and palp diameters. As predicted by the model, contact rates of the larger particles were approximately 6 times greater than that of the smaller particles (Fig 6). This figure also includes the contact rate ratio from the brittle star experiment (of Fig 5), taking the larger particle size as the peak in the distribution of captured particles (*D*_*p*,large_ ≈ 170*μ*m), and the smaller size as the peak in the final distribution of suspended particles (*D*_*p*,small_ ≈ 110*μ*m). Model predictions for the contact rate ratios of the larger and smaller particles in all experiments are again in excellent agreement with the experimental measurements (star in Fig 6).

**Fig 7:**
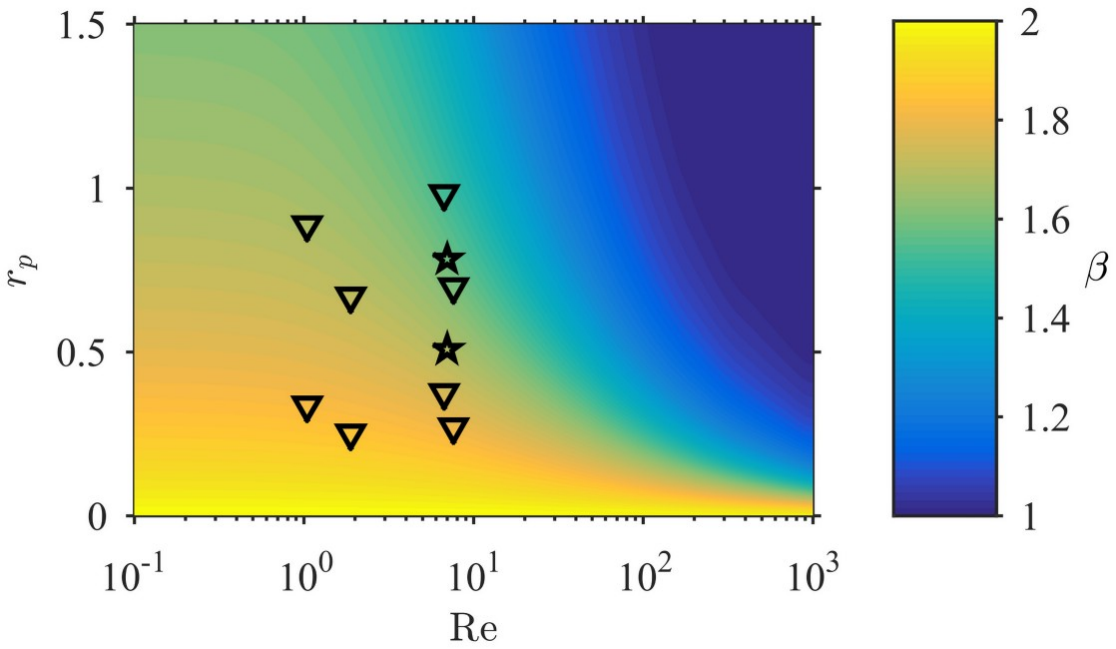
Model estimates of *β*, which governs the dependence of contact rate on particle size ($C\;R\propto {D_{p}}^{\beta }$), over the entire Re-*r*_*p*_ parameter space. The predicted variability (1 ≤ *β* ≤ 2) contrasts with the constant value of *β* = 2 from creeping flow and boundary layer theories (Eqs (4) and (5)). Markers represent the location of experimental data in the parameter space: (☆) peak sizes in distributions of captured (larger *r*_*p*_) and suspended (lower *r*_*p*_) particles in the brittle star experiment in Fig 5; (∇) the polychaetes experiments of [7].

The increase of contact rate with particle size is dictated by the local value of the exponent *β* in Eq (9) (i.e. $C\;R\propto {D_{p}}^{\beta }$). Theoretical models of contact that rely on small particle size suggest a constant value of *β* = 2 (Eqs (4) and (5)), a value supported by the empirical formulation of [14] (Eq (6)). However, this approximation is valid only for vanishing *r*_*p*_ [32]. For particles of finite size, local values of *β* are within the range 1 ≤ *β* ≤ 2 and decrease with relative particle size and Re (Fig 7). All experimental values of *β* deviate from the theoretical small-particle value of 2 and are within the range 1.4–1.9 (symbols in Fig 7). The particulate food of suspension feeders is usually large compared to the collector size (Table 2) and thus the dependence of contact rate on particle size typically falls outside the regime where the small particle size approximation holds.

This increase of *CR* with *D*_*p*_ can be understood through realisation that direct interception depends on the finite size of the particles [26, 28, 31, 32] (Fig 2). However, while large particles may contact collectors at greater rates, particle handling may limit the total rate of *capture*, as larger particles are more difficult to handle and retain [7].

### The variation of contact rate with collector diameter

**Fig 8:**
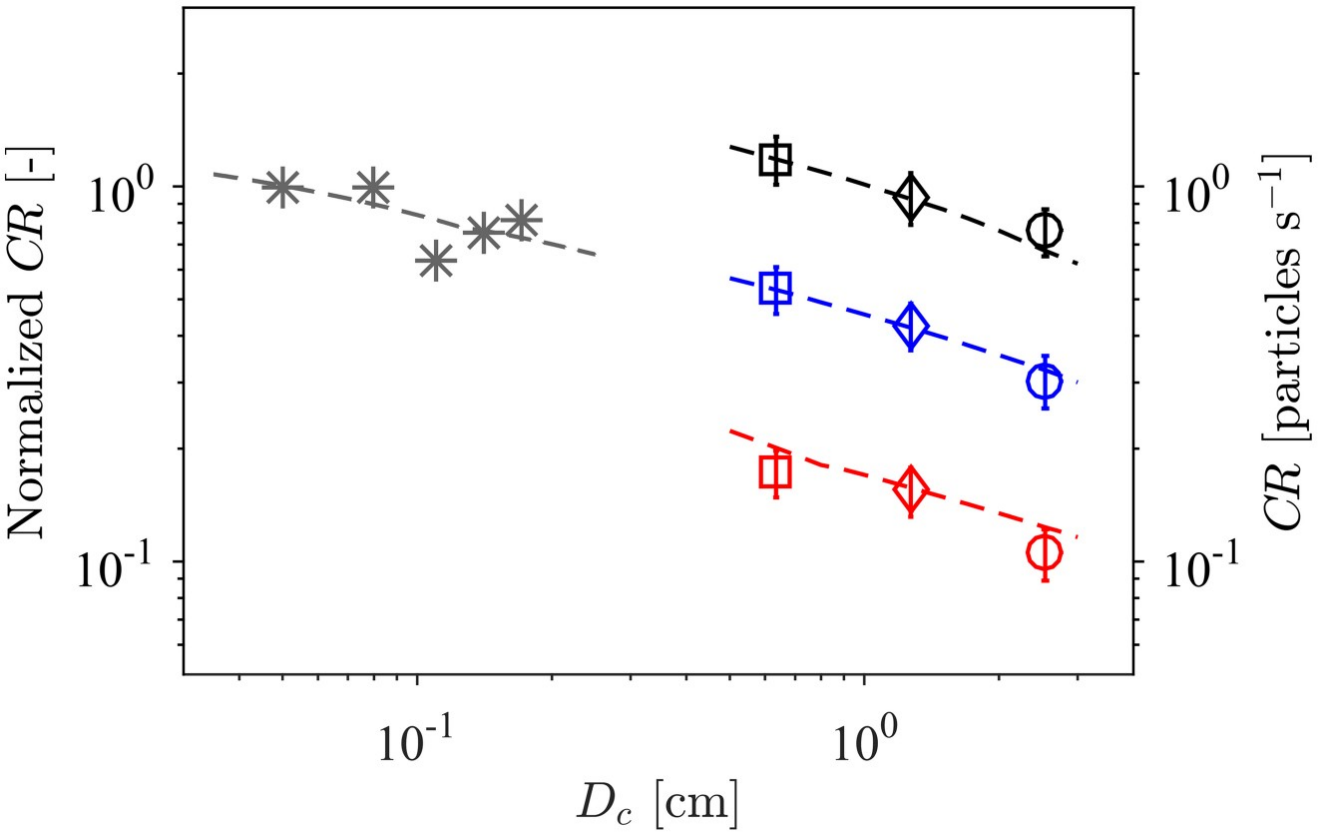
The observed decrease in contact rate with collector diameter. Coloured symbols represent experimental measurements of capture rate (on the right-hand axis) for rigid collectors at *U*_∞_ = 0.6 cm s^-1^ (red), 1.0 cm s^-1^ (blue) and 1.8 cm s^-1^ (black) [14] (markers as per Table 2). Gray asterisks (*) represent experimental measurements of normalized capture rate (on the left-hand axis) of bivalve spat by filamentous branched structures [13]; that is, the rate of contact with a collector of given diameter relative to that with the smallest collector employed. The broken lines indicate model estimates, which clearly support the reduction in particle contact with increasing collector size.

The effect of collector diameter (*D*_*c*_) on contact rate was analysed through experiments with (i) a branched collecting structure with a range of branch diameters [13] (Fig 1*C*) and (ii) cylindrical collectors where cylinder diameter was varied [14] (Fig 1*D*). Experimental results and numerical model predictions are in excellent agreement in showing the negative correlation between contact rate and collector diameter (Fig 8). That is, contact rate (counter-intuitively) decreases with increasing collector size. This implies that larvae and suspended material will contact and potentially settle at higher rates on finer structures than on larger structures (e.g. vegetation or engineered materials). Also, contact rates with food particles are higher on finer feeding appendages of suspension feeders which may have led to selection pressures towards thinner collectors, as discussed later.

**Fig 9:**
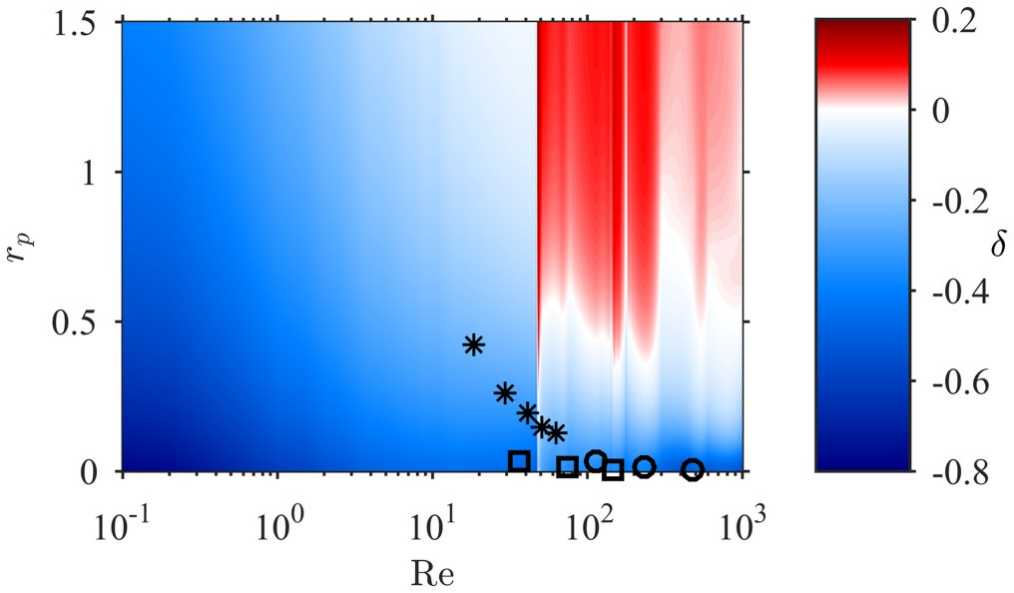
Numerical estimates of the exponent *δ* governing the dependence of contact rate on collector diameter ($C\;R\propto {D_{c}}^{\delta }$), over the entire Re-*r*_*p*_ parameter space. There are regions of both *δ* > 0 (increasing contact rate with collector size) and *δ* < 0 (decreasing contact rate with collector size). Markers represent the experimental data presented in Fig 8.

The overall relationship between contact rate and collector diameter is thus complex. On one hand, a larger collector enhances capture by increasing the collector Reynolds number (Eqs (4) and (5)) and the area over which ‘approaching’ particles can be sourced (Eq (1)). On the other, a larger collector diminishes capture by decreasing the relative particle size. The relationship of *CR* with *D*_*c*_ is determined by the local value of the exponent *δ* in Eq (9) (i.e. $C\;R\propto {D_{c}}^{\delta }$): for the contact rate to decrease with collector size, *δ* must be negative. Over the parameter range of the experiments, model predictions of *δ* were indeed negative (−0.45 < *δ* < −0.25). However, numerical predictions show regions of both negative (*δ* < 0) and positive (*δ* > 0) correlation within the full parameter space relevant to aquatic ecosystems (Fig 9). Positive correlation of *CR* with *D*_*c*_ occurs only for relatively large particles (*r_p_* ≳ 0.5) within the vortex shedding regime (Re > 47). Discontinuities in *δ* are evident after the flow regime abruptly transits into the vortex shedding regime (at Re = 47) with the first type of three-dimensional vortex shedding (Re = 180) and a second type of three-dimensional vortex shedding (Re = 260). The conditions for positive *δ* are common for many invertebrate suspension feeders (polychaetes, sea anemones, corals, brittle stars, crinoids), but only when capturing large particles such as macrozooplankton or organic-mineral aggregates (e.g. Table 2).

#### Optimal size of collecting structures

The complex relationship between contact rate and collector size may have strong implications for selective pressures in the evolution of suspension feeders’ morphology. [3] showed that the distribution of collector sizes is bimodal across protozoans, invertebrates, and vertebrates, with peaks at 0.2 *μ*m (e.g. cilia or fine-mesh elements) and 90 *μ*m (e.g. tentacles or elements of large filtering combs). Based on observations in the steady flow regime (Re < 47), it was suggested that the peak at 90 *μ*m was a consequence of natural selection towards animals with larger collectors that would benefit from operating at higher Reynolds numbers. However, we show here that any enlargement of collectors (e.g. forming compound cilia or thickening mesh elements) serves only to reduce contact rate in the steady flow regime because *CR* and *D*_*c*_ are always negatively correlated in this part of the parameter space (Fig 9). In this flow regime, this implies that contact rate always selects for a smaller collector diameter. This conclusion is not wholly restricted to the steady flow regime, as negative correlations between *CR* and *D*_*c*_ exist in the majority of the parameter space (Fig 9).

**Fig 10:**
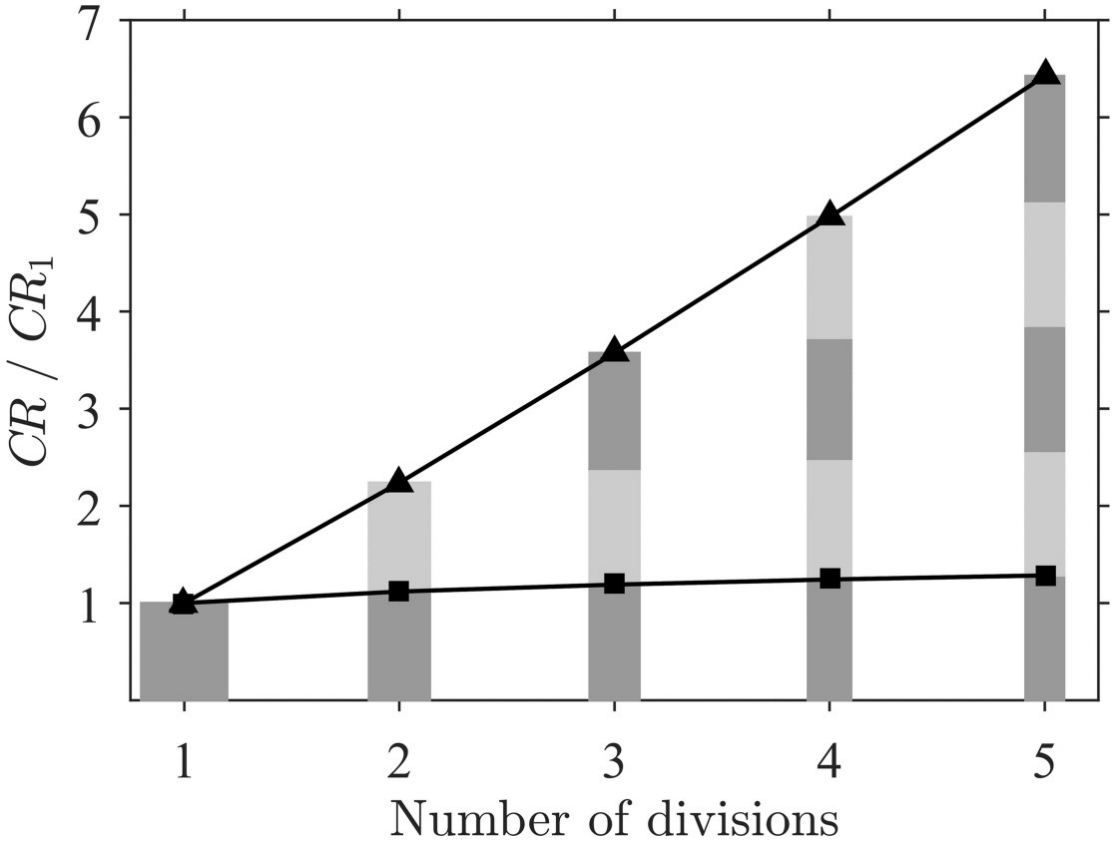
The advantage of a group of multiple cylindrical collectors over a single collector with the same total biomass. Triangles (▲) represent the total contact rate with the group of collectors (*CR*) relative to that of a single collector (*CR*_1_) with the same total biomass, and demonstrate a dramatic increase in total contact rate with the number of collectors in the group. Squares (■) show the minor contribution arising from the increase in contact rate with individual collectors within the group (due to increasing *r*_*p*_); this effect is small relative to the increase in frontal area with each division. The width of the bars indicates the change in collector diameter and the shading the number of collecting structures (while keeping total biomass constant).

These effects may have also generated selection pressures towards multiple (or longer) thin collectors (e.g. the arrays of filtering elements in many crustaceans, polychaetes, and echinoderms). As an example, consider a single suspension feeding collector with *D*_*c*_ = 100 *μ*m (and fixed height), capturing particles of *D*_*p*_ = 25 *μ*m in a velocity of *U*_∞_ = 6 cm s^-1^ (well within the ranges typically experienced by suspension feeders, Table 2). Distribution of the biomass of this collector among multiple collectors of the same height results in a dramatic increase in the total contact rate (Fig 10). This increase in contact rate is due partially to the increase in *CR* for each individual collector with decreasing collector size (squares in Fig 10), but is primarily due to the enhancement of frontal area created by biomass division. Under the condition of constant biomass, it can be inferred that $C\;R\propto {D_{c}}^{\delta -2}$, which implies a negative correlation (i.e. greater total contact with increasing subdivision) over the entire parameter space.

Natural selection for thinner collectors may be balanced, however, by other advantages of larger collectors such as improved retention and handling of particles after contact [7]; this may be responsible for maintaining the relatively large size of tentaculate feeding appendages (e.g. the 90 *μ*m mode found by [3]). Furthermore, larger diameters promote structural resistance of the collector and prevent pronation under strong flow conditions. The optimal collector size would then be the smallest diameter that allows organisms to optimally handle their preferred particulate food while also providing sufficient structural resistance under typical flow conditions.

## Conclusion

This paper has demonstrated the complex variation of particle capture in aquatic ecosystems, which are characterised by extensive ranges of system variables such as the fluid velocity, particle size and collector size. Importantly, the relationships between capture rate and these variables are not fixed, rather they vary significantly over the parameter space relevant to aquatic ecosystems. For example, although particle contact rate is inversely related to collector size in the parameter space studied by previous authors, we have shown that it increases with collector size when particles are relatively large and the flow regime involves vortex shedding. Thus, capture rates vary in opposite directions depending on an organism’s size, the nature of its diet or the sizes of particles being intercepted, and the range of flow speeds; thus creating different evolutionary selective pressures on morphology under various conditions. Specifically, maximizing capture rate favors thinner collectors in one range of ecologically relevant parameter space, but favours thicker collectors in another range. Furthermore, the variable functional relationships reveal that previous modeling with fixed relationships overestimated the dependence of contact rates on both velocity and particle size in important parts of the ecologically relevant parameter space. For example, suspension feeding rates and sedimentation rates do not always increase as strongly with velocity or particle size, as suggested previously. The numerical model presented here predicts rates of particle contact and their variability extremely accurately, even for complex biological structures, and it thus allows researchers to identify optimal values of key variables under specified scenarios. Full model output is provided here (in S1 Dataset) to allow quantitative prediction of the response of particle capture in ecosystems to changes in system conditions. The predictive numerical tool does not consider several potentially complicating features of real aquatic systems, including the effects of imperfect retention and collector flexibility. An understanding of these additional effects is required to optimize our capacity to quantitatively predict this critically important process in aquatic ecosystems.

## Supporting information

S1 Dataset The digital tool providing the output of all numerical simulations in this manuscript. (PDF)
