# Supplementary material for: On predicting particle capture rates in aquatic ecosystems
Source: PLoS One. 2021 Dec 22;16(12):e0261400. doi: 10.1371/journal.pone.0261400 (PMC8694431; doi:10.1371/journal.pone.0261400)
Supplement: S1 Dataset — (PDF) [file pone.0261400.s001.pdf]

Full dataset of model output

The full quantitative output of the numerical CFD model described in the main text is provided here through a digital tool developed in Python. Within the ranges $0 \le \mathrm{Re} \le 1000$ and $0 \le r_p \le 1.5$, the digital tool provides predictions of contact rate (CR) and contact efficiency (η) for inputted values of flow velocity (U_∞), particle and collector diameters (D_p and D_c, respectively), collector height (h_c), the concentration of particles in suspension (C_p) and the fluid kinematic viscosity (ν). Full instructions for installing the digital tool, and using it to predict rates of particle capture in aquatic ecosystems can be found here: http://github.com/alexisespinosa-research/pyCaptureDev

In previous work by this research team [28, 31, 32], values of the contact efficiency were presented graphically, as in Fig A.

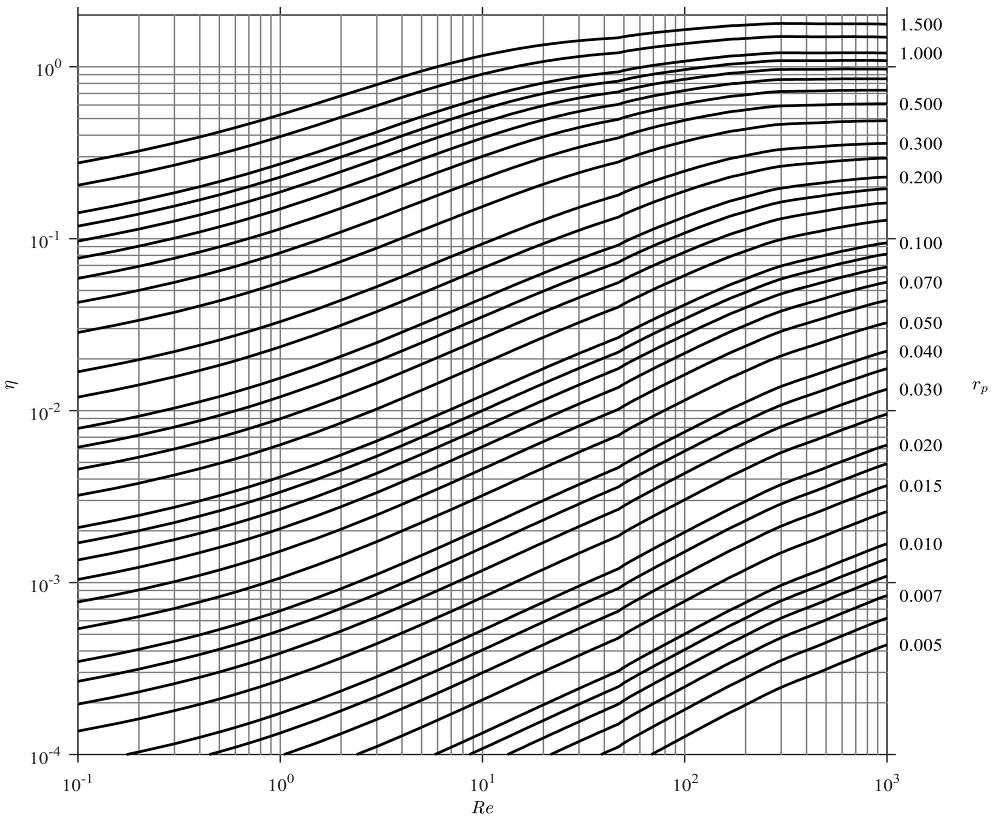

Fig A. The contact efficiency diagram for neutrally buoyant particles of finite size as a function of Reynolds number (Re) from the numerical analysis of particle capture of Espinosa-Gayosso *et al.* [31, 32]. Each curve corresponds to a particular value of the particle size ratio (r_p). Values of r_p are labelled on the right, with unlabelled lines at equal intervals of r_p.
